# S-substituted 2-mercaptoquinazolin-4(3H)-one and 4-ethylbenzensulfonamides act as potent and selective human carbonic anhydrase IX and XII inhibitors

**DOI:** 10.1080/14756366.2020.1742117

**Published:** 2020-03-19

**Authors:** Adel S. El-Azab, Alaa A.-M. Abdel-Aziz, Silvia Bua, Alessio Nocentini, Nawaf A. AlSaif, Mohammed M. Alanazi, Manal A. El-Gendy, Hany E. A. Ahmed, Claudiu T. Supuran

**Affiliations:** a Department of Pharmaceutical Chemistry, College of Pharmacy, King Saud University, Riyadh, Saudi Arabia; b Department of Neurofarba, Sezione di Scienze Farmaceutiche e Nutraceutiche, Università degli Studi di Firenze, Florence, Italy; c Department of Pharmaceutical Organic Chemistry, Faculty of Pharmacy, Al-Azhar University, Cairo, Egypt; d Pharmacognosy and Pharmaceutical Chemistry Department, College of Pharmacy, Taibah University, Al-Madinah Al-Munawarah, Saudi Arabia

**Keywords:** Metalloenzyme, quinazolinone incorporating ethylsulfonamide, selectivity CA inhibitors, molecular docking study

## Abstract

We evaluated the hCA (CA, EC 4.2.1.1) inhibitory activity of novel 4-(2-(2-substituted-thio-4-oxoquinazolin-3(4H)-yl)ethyl)benzenesulfonamides (compounds **2–20**) towards the isoforms I, II, IX, and XII. hCA Isoforms were effectively inhibited by most of new compounds comparable to those of AAZ. Compounds **2** and **4** showed interestingly efficient and selective antitumor (hCA IX and hCA XII) inhibitor activities (K_I_s; 40.7, 13.0, and 8.0, 10.8 nM, respectively). Compounds **4** and **5** showed selective hCA IX inhibitory activity over hCA I (SI; 95 and 24), hCA IX/hCA II (SI; 23 and 5.8) and selective hCA XII inhibitory activity over hCA I (SI; 70 and 44), hCA XII/hCA II, (SI; 17 and 10) respectively compared to AAZ. Compounds **12–17**, and **19–20** showed selective inhibitory activity towards hCA IX over hCA I and hCA II, with selectivity ranges of 27–195 and 3.2–19, respectively, while compounds **12**, **14–17**, and **19** exhibited selective inhibition towards hCA XII over hCA I and hCA II, with selectivity ratios of 48–158 and 5.4–31 respectively, compared to AAZ. Molecular docking analysis was carried out to investigate the selective interactions among the most active derivatives, **17** and **20** and hCAs isoenzymes. Compounds **17** and **20**, which are highly selective CA IX and XII inhibitors, exhibited excellent interaction within the putative binding site of both enzymes, comparable to the co-crystallized inhibitors.

**Highlights**
- Quinazoline-linked ethylbenzenesulfonamides inhibiting CA were synthesised.
- The new molecules potently inhibited the hCA isoforms I, II, IV, and IX.
- Compounds **4** and **5** were found to be selective hCA IX/hCA I and hCA IX/hCA II inhibitors.
- Compounds **4** and **5** were found to be selective hCA XII/hCA I and hCA XII/hCA II inhibitors.
- Compounds **12**–**17**, **19**, and **20** were found to be selective hCA IX/hCA I and hCA IX/hCA II inhibitors.
- Compounds **12**, **14**–**17**, **19** were found to be selective hCA XII/hCA I and hCA XII/hCA II inhibitors.

**Graphical Abstract**
Compounds **4** and **5** are selective hCA IX and XII inhibitors over hCA I (selectivity ratios of 95, 23, and 24, 5.8, respectively) and hCA II (selectivity ratios of 70, 17, and 44, 10 respectively). Compounds **12–17**, and **19–20** are selective hCA IX inhibitors over hCA I (selectivity ratios of 27-195) and hCA II (selectivity ratios of 3.2-19). Compounds **12**, **14–17** and **19** are also selective hCA XII inhibitors over hCA I (selectivity ratios of 48-158) and hCA II (selectivity ratios of 5.4-31).

## 1. Introduction

**Figure 1.**
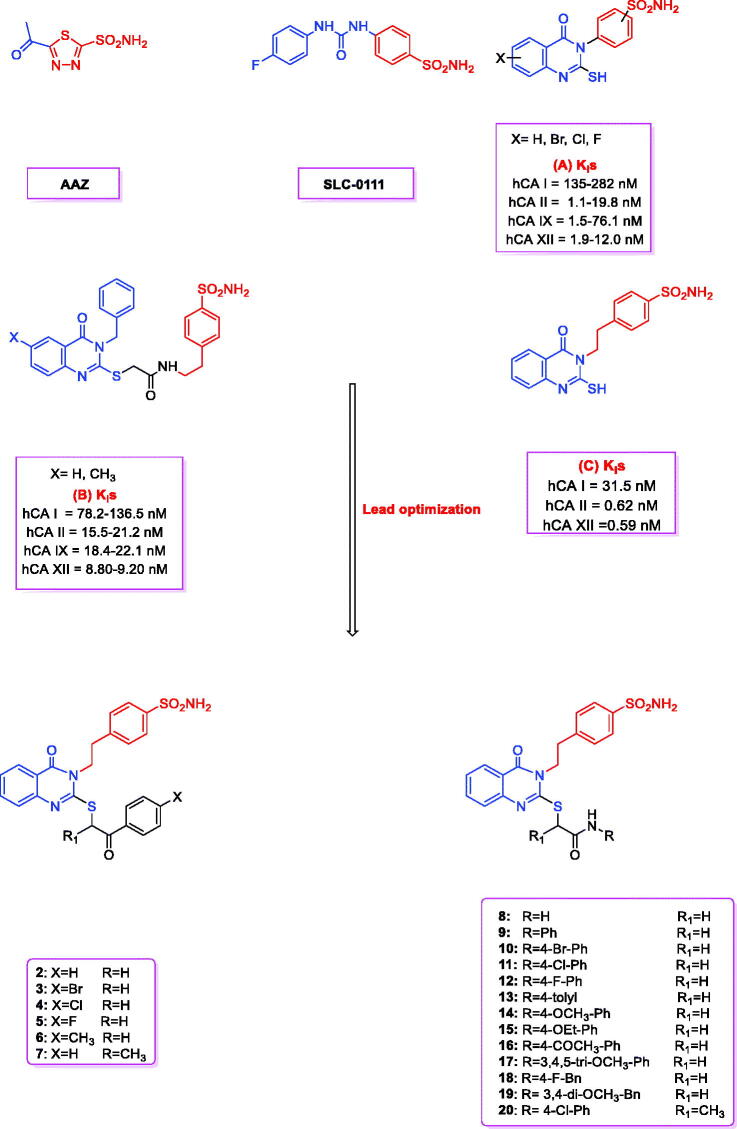
Structures of AAZ, SLC-0111, **A**–**C**, and the designed quinazoline derivatives (**2**–**20**) as CAIs.

Carbonic anhydrases (CAs; EC 4.2.1.1) constitute the superfamily of metalloenzymes that catalyse the CO_2_ hydration and dehydration reactions. CAs are classified into eight genetically distinct families, named α-, β-, γ-, δ-, ζ-, η-, ɵ- and ι-CAs^1^^,^^2^. 15 α-class CA isozymes have been detected in humans, which are further classified into four different subsets on the basis of their subcellular localisation—CA I, II, III, VII, VIII, X, XI, XIIII are cytosolic proteins, CA IV is a glycosylphosphatidylinositol (GPI)-anchored protein, CA VA and VB are located in the mitochondrial matrix, CA VI is secreted, and CA IX, XII and XIV are trans-membrane isoforms^1–3^. Human CAs (hCAs) are spread in the human body, and are implicated in a plethora of essential physiological processes. Therefore, the dysregulated expression and/or activity of the CAs can lead to various pathological conditions^2^. CA II is the most physiologically relevant CA isoform, implicated in various disorders including cerebral oedema, glaucoma (such as CA XII), and epilepsy. It is conversely off-target, as CA I, when targeting tumours where CA IX and XII are overexpressed and represent validated targets to combat the growth of both primary tumours and metastasis^4^^,^^5^. The high structural similarities between various CA isoforms necessitate high selectivity in the design of small-molecule anti-CA drugs for the treatment of diseases associated with CA dysregulation, to minimise the side effects^3^. Benzene sulphonamides are one of the best-known molecules clinically used as CA inhibitors. Additionally, “SLC-011 (Figure 1), a benzenesulfonamide, is a selective CA IX/XII inhibitor currently being evaluated in a Phase I trial for the treatment of solid, metastatic tumors”^6–10^. Sulphonamide derivatives are not only one of the most preferred CA inhibitor classes^9^^,^^11–23^, but also important COX-2 inhibitors and antitumor agents^17^^,^^19^^,^^24–26^. The quinazolinone scaffold is also used widely across medicinal chemistry^27–43^. (6-Iodo or 7-flouro-2-merqapto-4-(3H)-quinazolinone3-yl)-benzenesulfonamides (**A**, Figure 1) have been shown to potently inhibit CA I, II, IX, and XII^44^^,^^45^. A number of 2-((3-benzyl-4-oxo-3,4-dihydroquinazolin-2-yl)thio)-*N*-(4-sulfamoylphenethyl)anildes (**B**, Figure 1) also showed potent inhibitory activity against different hCA isoforms^38^. The 2-mercapto-4(3H)-quinazolinone derivatives containing ethylsulfonamide tail (**C**, Figure 1) showed strong inhibitory activity against different hCA isoforms with low-concentration inhibition constants

Here, we studied 2-mercaptoquinazolinone, (**C**, Figure 1) a slightly polar and non-selective hCA inhibitor. Because the sulfhydryl group has been reported to be associated with various metabolic and pharmacological problems^46–49^, we used a 2-mercaptoquinazolinone scaffold bearing an ethylsulfonamide head with alkylation of the thione group with a terminal lipophilic moiety, so that it can interact selectively with CA through both, hydrogen and hydrophobic interactions. Here, we synthesised various derivatives of 2-mercaptoquinazolinone (**2–20**, Figure 1) with different selectivity criteria for the hCA inhibitors, particularly for the tumor-associated hCA IX and hCA XII. The role of alkyl substituent in 2-mercaptoquinazolinone was computationally analysed and the conserved residues responsible for the target selectivity were identified.

## 2. Materials and methods

### 2.1. Chemistry

Melting points were recorded on a Barnstead 9100 electrothermal melting point apparatus (UK). IR spectra (KBr) were recorded on a FT-IR Perkin-Elmer spectrometer (Perkin Elmer Inc., MA). NMR (^1^H and ^13^C NMR) spectra were recorded with Bruker 700 MHz spectrometers (Zurich, Switzerland). Micro-analytical data (C, H, and N) were obtained using a Perkin-Elmer 240 analyser (Perkin Elmer Inc., MA) and agreed with the proposed structures within ±0.4% of the theoretical values. Mass spectra were recorded on a Varian TQ 320 GC/MS/MS mass spectrometer (Varian, Palo Alto, CA). Thione **1** and compounds **8–20** were prepared as described earlier^50^^,^^51^.

#### 2.1.1. General procedure for synthesis of 4-(2-(2-((2-(4-substituted-phenyl)-2-oxoethyl)thio)-4-oxoquinazolin-3(4H)-yl)ethyl)benzenesulfonamide (2–7)

A mixture of thione **1** (1 mmol, 361 mg) and potassium carbonate (3 mmol, 415 mg) in 6 ml acetone were stirred at room temperature for one hour. Appropriate phenacyl bromide (1 mmol) was added and the reaction mixture was stirred at room temperature for 9–12 h, filtered, and the crude solid was washed with water, dried and recrystallized from ethanol (^1^H & ^13^C NMR supplementary material).

*4-(2-(4-Oxo-2-((2-oxo-2-phenylethyl)thio)quinazolin-3(4H)-yl)ethyl)benzenesulfonamide (2)*: m.p 246–247°; 94% yield; IR (KBr, cm^−1^) *ν*: 3284, 3237 (NH), 1665 (C=O), 1342, 1151 (O=S=O); ^1^H NMR (700 MHz, DMSO-d_6_): *δ* 8.14 (t, 2H, *J* = 7.14 and 1.26 Hz), 8.04 (dd, 1H, *J* = 7.91 and 1.26 Hz), 7.82 (d, 2H, *J* = 8.26 Hz), 7.74 (t, 1H, *J* = 7.49 Hz), 7.66 (t, 1H, *J* = 16.71 and 6.96 Hz), 7.62 (t, 2H, *J* = 7.80 and 7.77 Hz), 7.52 (d, 2H, *J* = 8.26 Hz), 7.41 (t, 1H, *J* = 7.17 Hz), 7.37 (s, 2H), 6.98 (d, 1H, *J* = 8.12 Hz), 4.92 (s, 2H), 4.33 (t, 2H, *J* = 16.25 Hz), 3.14 (t, 2H, *J* = 16.20 Hz); ^13^C NMR (176 MHz, DMSO-d_6_): *δ* 194.04, 160.76, 156.10, 146.92, 143.11, 142.29, 136.90, 135.19, 134.01, 129.67, 129.29, 128.79, 126.92, 126.45, 125.87, 119.08, 45.69, 39.38, 33.67; Ms; *m/z* (479).

*4-(2-(2-((2-(4-Bromophenyl)-2-oxoethyl)thio)-4-oxoquinazolin-3(4H)-yl)ethyl)benzenesulfonamide (3)*: m.p 248–248°; 95% yield; IR (KBr, cm^−1^) *ν*: 3280, 3236 (NH), 1686 (C=O), 1340, 1153 (O=S=O); ^1^H NMR (700 MHz, DMSO-d_6_): *δ* 8.07 (d, 2H, *J* = 8.26 Hz), 8.04 (d, 1H, *J* = 7.84 Hz), 8.85 (d, 2H, *J* = 8.19 Hz), 7.81 (d, 2H, *J* = 7.98 Hz), 7.68 (t, 1H, *J* = 7.63 Hz), 7.52 (d, 2H, *J* = 8.05 Hz), 7.41 (d, 1H, *J* = 7.45 Hz), 7.37 (s, 2H), 7.00 (d, 1H, *J* = 8.19 Hz), 4.89 (s, 2H), 4.32 (t, 2H, *J* = 16.05 Hz), 3.13 (t, 2H, *J* = 16.04 Hz); ^13 ^C NMR (176 MHz, DMSO-d_6_): *δ* 193.41, 160.74, 156.02, 146.88, 143.12, 142.27, 135.9266, 135.26, 132.38, 130.81, 129.67, 128.10, 126.93, 126.55, 126.52, 126.45, 125.87, 119.08, 45.72, 39.27, 33.67; Ms; 558.0; Ms; (*m/z*; 557, M + 2; 559).

*4-(2-(2-((2-(4-Chlorophenyl)-2-oxoethyl)thio)-4-oxoquinazolin-3(4H)-yl)ethyl)benzenesulfonamide (4)*: m.p 250–251°; 93% yield; IR (KBr, cm^−1^) *ν*: 3281, 3239 (NH), 1684 (C=O), 1345, 1159 (O=S=O); ^1^H NMR (700 MHz, DMSO-d_6_): *δ* 8.15 (d, 2H, *J* = 8.43 Hz), 8.04 (d, 1H, *J* = 7.85 Hz), 7.82 (d, 2H, *J* = 8.05 Hz), 7.70 (d, 2H, *J* = 8.40 Hz), 7.67 (d, 1H, *J* = 7.05 Hz), 7.52 (d, 2H, *J* = 8.05 Hz), 7.41 (t, 1H, *J* = 7.45 Hz), 7.37 (s, 2H), 6.99 (d, 1H, *J* = 8.19 Hz), 4.89 (s, 2H), 4.32 (t, 2H, *J* = 16.09 Hz), 3.13 (t, 2H, *J* = 16.06 Hz); ^13 ^C NMR (176 MHz, DMSO-d_6_): *δ* 193.20, 160.74, 156.03, 146.89, 143.12, 142.28, 138.89, 135.60, 135.25, 130.73, 129.67, 129.43, 126.93, 126.53, 125.86, 119.08, 45.72, 39.28, 33.67; Ms; 514; Ms; (*m/z*; 513, M + 1; 514).

*4-(2-(2-((2-(4-Fluorophenyl)-2-oxoethyl)thio)-4-oxoquinazolin-3(4H)-yl)ethyl)benzenesulfonamide (5)*: m.p 249–250°; 92% yield; IR (KBr, cm^−1^) *ν*: 3278, 3238(NH), 1666 (C=O), 1342, 1152 (O=S=O); ^1^H NMR (700 MHz, DMSO-d_6_): *δ* 8.23 (dd, 2H, *J* = 13.95 and 2.66 Hz), 8.04 (d, 1H, *J* = 7.84 Hz), 7 82 (d, 2H, *J* = 8.05 Hz), 7.68 (t, 1H, *J* = 7.63 Hz), 7.52 (d, 2H, *J* = 7.98 Hz), 7.46 (t, 2H, *J* = 8.71 Hz), 7.41 (t, 1H, *J* = 7.49 Hz), 7.37 (s, 2H), 6.99 (d, 1H, *J* = 8.12 Hz), 4.90 (s, 2H), 4.33 (t, 2H, *J* = 16.09 Hz), 3.14 (t, 2H, *J* = 16.06 Hz); ^13 ^C NMR (176 MHz, DMSO-d_6_): *δ* 192.73, 166.36, 164.93, 160.7571, 156.07, 146.89, 143.12, 142.28, 135.24, 133.65, 133.64, 131.88, 131.83, 129.67, 126.92, 126.52, 125.87, 119.08, 116.41, 116.29, 45.70, 39.26, 33.67; Ms; *m/z* (497).

*4-(2-(4-Oxo-2-((2-oxo-2-(p-tolyl)ethyl)thio)quinazolin-3(4H)-yl)ethyl)benzenesulfonamide (6)*: m.p 257–258°; 92% yield; IR (KBr, cm^−1^) *ν*: 3281, 3237 (NH), 1665 (C=O), 1339, 1150 (O=S=O); ^1^H NMR (700 MHz, DMSO-d_6_): *δ* 8.04 (t, 3H, *J* = 7.12 and 4.90 Hz), 7.82 (d, 2H, *J* = 7.75 Hz), 7.68 (t, 1H, *J* = 7.59 Hz), 7.52 (d, 2H, *J* = 7.84 Hz), 7.42 (t, 3H, *J* = 7.84 and 10.71 Hz), 7.37 (s, 2H), 7.04 (d, 1H, *J* = 8.19 Hz), 4.90 (s, 2H), 4.33 (t, 2H, *J* = 15.79 Hz), 3.14 (t, 2H, *J* = 15.79 Hz), 2.44 (s, 3H); ^13 ^C NMR (176 MHz, DMSO-d_6_): *δ* 193.40, 160.78, 156.12, 146.94, 144.46, 143.12, 142.30, 135.21, 134.33, 129.83, 129.67, 128.93, 126.91, 126.52, 125.95, 119.09, 45.65, 39.41, 33.67, 21.73; Ms; *m/z* (493).

*4-(2-(4-Oxo-2-((1-oxo-1-phenylpropan-2-yl)thio)quinazolin-3(4H)-yl)ethyl)benzenesulfonamide (****7****)*: m.p 245–246°; 90% yield; IR (KBr, cm^−1^) *ν*: 3279, 3237 (NH), 1668 (C=O), 1347, 1154 (O=S=O); ^1^H NMR (700 MHz, DMSO-d_6_): *δ* 8.16 (d, 2H, *J* = 7.76 Hz), 8.29 (d, 1H, *J* = 7.85 Hz), 7.80 (d, 2H, *J* = 7.84 Hz), 7.73 (t, 1H, *J* = 7.31 Hz), 7.65–7.61 (m, 3H), 7.84 (d, 2H, *J* = 7.84 Hz), 7.39 (t, 1H, *J* = 7.52 Hz), 7.37 (s, 2H), 6.78 (d, 1H, *J* = 8.12 Hz), 5.75 (q, 1H, *J* = 7.16 Hz), 4.29–4.19 (m, 2H), 3.07 (t, 2H, t, *J* = 12.58 Hz), 1.57 (d, 3H, *J* = 7.19 Hz); ^13 ^C NMR (176 MHz, DMSO-d_6_): *δ* 198.20, 160.67, 155.81, 146.87, 143.12, 142.23, 135.98, 135.15, 134.01, 129.69, 129.34, 128.95, 126.92, 126.57, 126.48, 125.46, 119.13, 46.23, 45.76, 33.60, 16.44; Ms; 493.00; Ms; *m/z* (493).

### 2.2. CA inhibition

The hCA I, II, IX, and XII isoenzyme inhibition assays were performed according to the reported method using the SX.18 MV-R stopped-flow instrument (Applied Photophysics, Oxford, UK)^52–54^. All CA isoforms were recombinant isoforms obtained in-house, as reported earlier^55^^,^^56^.

### 2.3. Molecular docking method

The molecular docking protocol was conducted according to the reported methods^28^^,^^32^^,^^33^^,^^41–43^^,^^57–64^ using MOE 2008.10 from the Chemical Computing Group Inc^65^. The crystal structures of CA-IX (PDB ID: 5FL4) and CA-XII (PDB ID: 1JCZ) were obtained from the protein data bank^66^^,^^67^.

## 3. Results and discussion

### 3.1. Chemistry

**Scheme 1.**
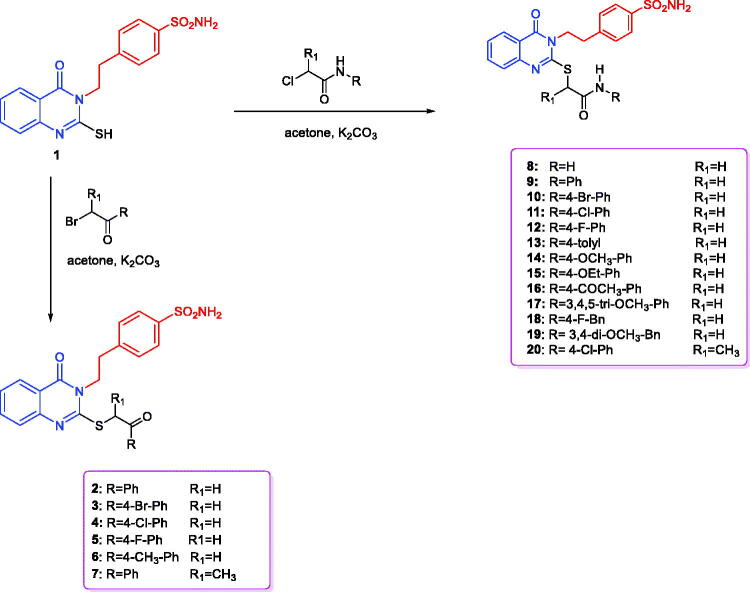
Synthesis of the designed quinazoline derivatives (**2**–**20**).

4-(2-(4-Oxo-2-thioxo-1,4-dihydroquinazolin-3(2*H*)-yl)ethyl)benzenesulfonamide (**1**) was obtained *via* the reaction of 4-(2-isothiocyanatoethyl)benzenesulfonamide, triethylamine and 2-aminobenzoic acid in boiling ethanol^50^^,^^51^ (Scheme 1). Stirring of compound **1** with potassium carbonate in acetone and different phenacyl bromides produced the corresponding 4-(2-(2-((2-(4-substituted-phenyl)-2-oxoethyl)thio)-4-oxoquinazolin-3(4*H*)-yl)ethyl)benzenesulfonamides **2–7** with 90–95% yield. Various spectroscopic studies were conducted to validate the structures of the newly synthesised compounds, **2–7**. The target compounds, **2–6**, were validated by the diminishing of the thioamidic proton (NH–C=S) at 13.03 ppm and that of the thione moiety (NH–C=S) at 175.29 ppm, as well as by the presence of the phenacyl carbonyl group (SCH_2_COAr) at 194.04–192.73 ppm, with singlet peaks at 4.92–4.89 ppm and 39.41–39.26 ppm due to the phenacyl (SCH_2_COAr) moiety, in the ^1^H and ^13^C NMR spectra, respectively. Additionally, 4-(2-(4-oxo-2-((1-oxo-1-phenylpropan-2-yl)thio)quinazolin-3(4*H*)-yl)ethyl)benzenesulfonamide (**7**) was confirmed by presence of the carbonyl group of (S(CH)CH_3_COAr) at 198.20 ppm in the ^13^C NMR spectrum, as well as by the quartette (S(CH)CH_3_COAr) and doublet (S(CH)CH_3_COAr) peaks at 5.75 and 1.57 ppm respectively in the ^1^H NMR spectrum, together with the characteristic peaks (S(CH)CH_3_COAr) at 46.23 and (S(CH)CH_3_COAr) at 16.44 ppm in the ^13^C NMR spectrum. The ethylbenzenesulfonamide amino group (NH_2_) (in compounds **2–7**) was long-established by the presence of a typical singlet peak at 7.37 ppm in the ^1^H NMR spectrum. The tails of aliphatic ethylbenzenesulfonamide moiety were fixed by triplet peaks at 4.33–4.32 and 3.15–3.12 ppm in the ^1^H NMR spectrum and distinctive peaks at 45.72–45.65 and 33.67 ppm in ^13^C NMR spectrum, respectively. 2-Substituted mercapto-4(3*H*)-quinazolinones (**8–20**) were prepared in 90–96% yield by mixing compound **1** and 2-chloro-*N*-substitutedamide in acetone at room temperature in the presence of potassium carbonate^51^.

### 3.2. CA inhibitory activity

The CA inhibitory activity of 4-(2-(2-(substituted-thio)-4(3H)-quinazolinon-3-yl)ethyl)benzenesulfonamides (compounds **2–20**) towards hCA I, II, IV, and IX isoforms was measured and compared to acetazolamide (AAZ), a typical sulphonamide inhibitor. hCA I was effectively inhibited by compounds **2** and **4–13** with the inhibition-constant (K_I_) values ranging from 114.5–938.3 nM (AAZ: K_I_ = 250.0 nM). Compounds **3** and **16** showed moderate activity with K_I_ values of 1447.0 and 1697.0 nM, respectively, while compounds **14–15** and **17–20** showed weak activity with K_I_ values ranging from 2048–5467 nM. Compounds **5**, **8**, **9**, **11**, **12**, and **20** were verified to be effective hCA II inhibitors, with K_I_ values of 25.4–95.4 nM (AAZ: K_I_ = 12.0 nM). Compounds **2**, **3**, **4**, **6**, **7**, **10**, **14**, and **16** showed modest hCA II inhibitory activity with K_I_ values ranging between 116.2 and 266.1 nM, whereas compounds **13** and **15** showed a weak inhibitory activity with K_I_ values of 304.6 and 1099.0 nM, respectively. Compounds **2–17** and **20** displayed potent hCA IX inhibitory activity with K_I_ values ranging from 8.0 to 100.4 nM, which were greater than or nearly identical to that of AAZ (K_I_ = 25.0 nM), whereas compounds **18** and **19** showed modest hCA IX inhibitory activity with K_I_ values ranging between 256.4 and 145.1 nM, respectively. 4-(2-(2-(Substituted-thio)-4(3H)-quinazolinon-3-yl)ethyl)benzenesulfonamide derivatives **2**, **4**, **5**, **8**, **9**, **11**, **12**, **13**, **14**, **16** and **17** showed potent hCA XII inhibitory activity with K_I_ values of 2.4–49.1 nM compared to AAZ (K_I_ = 5.7 nM), whereas compounds **3**, **6**, **7**, **10**, **15**, **18**, **19**, and **20** exerted moderate hCA XII inhibitory activities with K_I_ values of 59.7–113.4 nM (Table 1). On the other hand, the selectivity factor is critical goal to increase the value of the new synthesised compounds. New compounds, such as **2** and **4** showed characteristic effective and selective antitumor (hCA IX and hCA XII) carbonic anhydrase inhibitory activity with K_I_ values (compound **2**; 40.7 and 13.0 nM) and K_I_ values (compound **4**; 8.0, and 10.8 nM) compared with AAZ (K_I_ values of 25 and 5.7 respectively). 4-(2-(2-((2-(4-Substituted-phenyl)-2-oxoethyl)thio)-4-oxoquinazolin-3(4*H*)-yl)ethyl)benzenesulfonamides (compounds **2–7**) showed high selectivity in the inhibition of hCA IX over hCA I and hCA II (in the range of 15.0–95.0 and 2.3–23.0, respectively), as well as selectivity in the inhibition of hCA XII over hCA I and hCA II (in the range of 5.5–70.0 and 2.5–17.0, respectively) (Table 1).

**Table 1.** Inhibition data of hCA isoforms hCA I, II, IX and XII for sulphonamides using AAZ as standard drug. 
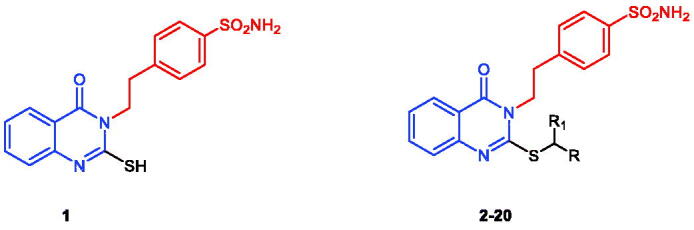

Comps	R	R_1_	K_I_ (nM)^a^				Selectivity analysis			
			hCA I	hCA II	hCA IX	hCA XII	hCA I/IX	hCA I/XII	hCA II/IX	hCA II/XII
**1**	–	–	31.5	0.62	–	0.59	–	53.12	–	1.05
**2**	COPh	H	592.7	140.8	40.7	13.0	15	46	3.5	11
**3**	CO(4-Br-Ph)	H	1447	174.7	75.2	69.6	19	21	2.3	2.5
**4**	CO(4-Cl-Ph)	H	758.7	186.6	8.0	10.8	95	70	23	17
**5**	CO(4-F-Ph)	H	399.5	95.4	16.5	9.1	24	44	5.8	10
**6**	CO(4-CH_3_-Ph)	H	471.0	116.2	25.1	85.1	19	5.5	4.6	1.4
**7**	CO(4-Br-Ph)	CH_3_	978.3	202.6	63.2	76.8	15	13	3.2	2.6
**8**	CONH_2_	H	114.5	25.4	34.5	2.4	3.3	48	0.7	11
**9**	CONHPh	H	459.7	69.7	27.3	38.4	17	12	2.6	1.8
**10**	CONH(4-Br-Ph)	H	697.1	119.3	64.9	61.0	11	11	1.8	2
**11**	CONH(4-Cl-Ph)	H	726.4	92.0	66.8	31.6	11	23	1.4	2.9
**12**	CONH(4-F-Ph)	H	548.6	87.6	12.7	8.7	43	63	6.9	10
**13**	CONH(4-CH_3_-Ph)	H	878.1	304.6	37.4	45.2	23	19	8.1	6.7
**14**	CONH(4-OCH_3_-Ph)	H	2567	266.1	84.0	49.1	31	52	3.2	5.4
**15**	CONH(4-OC_2_H_5_-Ph)	H	3654	684.2	35.9	59.7	102	61	19	11
**16**	CONH(4-COCH_3_-Ph)	H	1697	200.1	24.1	22.5	70	75	8.3	8.9
**17**	CONH(3,4,5-tri-OCH_3_-Ph)	H	2672	519.4	100.4	16.9	27	158	5.2	31
**18**	CONH(4-F-Bn)	H	2048	975.4	256.4	113.4	8	18	3.8	8.6
**19**	CONH(3,4-diOCH_3_-Bn)	H	5467	1099	145.1	97.3	38	56	7.6	11
**20**	CONH(4-Cl-Ph)	CH_3_	3628	75.4	18.6	66.7	195	54	4.1	1.1
**AAZ**	–		250.0	12.0	25.0	5.7	10	44	0.5	2.1

^a^ Mean from 3 different assays, obtained using a stopped flow technique (errors were in the range of ±5–10% of the reported values).

Compounds **4** and **5** showed high selectivity in the inhibition of hCA IX over hCA I and hCA II, with selectivity ratios of 95.0 and 23.0, respectively for compound **4**, and those of 24.0 and 5.8, respectively, for compound **5**, compared with AAZ selectivity ratios of 10.0 and 0.5, respectively. Additionally, compounds **4** and **5** showed selective inhibition of hCA XII over hCA I and hCA II with selectivity ratios of 70.0 and 17.0, respectively, for compound **4**, and 44.0 and 10.0, respectively, for compound **5**, compared with AAZ selectivity ratios of 44.0 and 2.1, respectively. *N*-(substituted)-2-((4-oxo-3-(4-sulfamoylphenethyl)-3,4-dihydroquinazolin-2-yl)thio)amides (compounds **8–20**) showed high selectivity in the inhibition of hCA IX over hCA I and hCA II, with selectivity ratios in the range 3.3–195.0 and 0.7–8.3 respectively, compared with AAZ selectivity ratios of 10.0 and 0.5 respectively, and that of hCA XII over hCA I and hCA II, respectively with selectivity ratios in the range 11.0–158.0 and 1.8–31.0, compared with AAZ selectivity ratios of 44.0 and 2.1 respectively. Compounds **12**–**17**, and **19–20** showed selective inhibition of hCA IX over hCA I and hCA II, with selectivity ratios of 23.0–195.0 and 3.2–19.0, respectively, compared with AAZ selectivity ratios of 10.0 and 0.5. Additionally, Compounds **12**, **14**–**17**, and **19** showed selective inhibition of hCA XII over hCA I and hCA II, with selectivity ratios of 48.0–158.0 and 5.4–31.0, respectively, compared with AAZ selectivity ratios of 44.0 and 2.1 respectively.

### 3.3. Structure-activity relationship (SAR) analysis

Several synthesised quinazolinone derivatives (compounds **2**–**20**) were potent inhibitors of the hCA isoforms.

#### 3.3.1. SAR analysis of hCA I inhibition

SAR analysis of hCA I inhibition indicated revealed several key features. (1) 4–(2-(4-Oxo-2-((2-oxo-2-phenylethyl)thio)quinazolin-3(4H)-yl)ethyl)benzenesulfonamide (**2**), with a K_I_ value of 592.7 nM, was more potent than 4-(2-(2-((1-(4-substituted-phenyl)-1-oxopropan-2-yl)thio)-4-oxoquinazolin-3(4*H*)-yl)ethyl)benzenesulfonamides **3**–**4** and 4-(2-(4-oxo-2-((1-oxo-1-phenylpropan-2-yl)thio)quinazolin-3(4*H*)-yl)ethyl)benzenesulfonamide **7**, with K_I_ values of 758.7–1447 nM, but less potent than 4-(2-(2-((1-(4-flouro/4-methyl-phenyl)-1-oxopropan-2-yl)thio)-4-oxoquinazolin-3(4*H*)-yl)ethyl)benzenesulfonamide **5** and **6** with K_I_ values of 399.5–471.0 nM. (2) Unsubstituted-*N*-acetamide **8** (K_I_ value = 114.5 nM) was more active than the corresponding *N*-phenylacetamide **9** (K_I_ value = 459.7 nM. (3) Substitution of the phenyl ring of *N*-phenylacetamide **9** (K_I_ value = 459.7 nM) resulted in substituted-*N*-phenylacetamides **10**–**17** and *N*-phenylpropanamide **20** with significantly decreased CA inhibitory activity (K_I_ values = 548.6–3654 nM). (4) The hCA I inhibitory activity of *N*-(4-fluorophenyl)-2-((4-oxo-3-(4-sulfamoylphenethyl)-3,4-dihydroquinazolin-2-yl)thio)acetamide (**12**), with a K_I_ value of 548.6 nM, was more stronger than the corresponding *N*-(4-fluorobenzyl)-2-((4-oxo-3-(4-sulfamoylphenethyl)-3,4-dihydroquinazolin-2-yl)thio)acetamide (**18**) K_I_ of 2048 nM. (5) hCA I inhibition of *N*-acetamide **11**, with a K_I_ value of 726.4 nM, was more powerful than the corresponding *N*-propanamide **20** with a K_I_ value of 3628 nM.

#### 3.3.2. SAR analysis for hCA II inhibition

The SAR analysis for hCA II inhibition revealed several key features. (1) 4-(2-(2-((1-(4-Fluorophenyl/4-methylphenyl)-1-oxopropan-2-yl)thio)-4-oxoquinazolin-3(4*H*)-yl)ethyl)benzenesulfonamides **5** and **6** with K_I_ values of 95.4–116.2 nM were more effective than unsubstituted phenyl and other substituted phenyl derivatives, such as compounds **2**–**4** and **7** with K_I_ values of 140.8–202.6 nM. (2) hCA II inhibition of 2-((2-oxo-2-phenylethyl)thio)quinazolinone **2,** with a K_I_ value of 140.8 µM, was stronger than the corresponding 2-((1-oxo-1-phenylpropan-2-yl)thio)quinazolinone **7** with a K_I_ value of 202.6 nM. (3) *N*-Phenylacetamide **9** with a K_I_ value of 69.7 nM was less potent than unsubstituted-*N*-acetamide **8** (K_I_ value = 25.4 nM). (4) Substitution of the phenyl ring of *N*-phenylacetamide **9** (K_I_; 69.7 nM) resulted in substituted-*N*-phenylacetamides **10**–**17** and *N*-phenylpropanamide **20** with considerably diminished CA II inhibitory activity (K_I_ values of 75.4–684.2 nM); (5) The hCA II inhibitory effect of *N*-acetamide **11** (K_I_ value = 92 nM) was less potent than the corresponding *N*-propanamide **20** (K_I_ value = 75.4 nM). (6) The hCA II inhibitory activity of *N*-(4-fluorophenyl)acetamide **12**, with a K_I_ value of 87.6 nM, was stronger than the corresponding *N*-(4-fluorobenzyl)acetamide **18** (K_I_ of 2048 nM).

#### 3.3.3. SAR analysis of hCA IX inhibition

SAR analysis of hCA IX inhibition revealed several key factors. (1) The 2-((2-oxo-2-phenylethyl)thio)quinazolinone **2**, with a K_I_ value of 40.7 nM, was more potent than 2-((1-oxo-1-phenylpropan-2-yl)thio)quinazolinone **7** with K_I_ value of 63.2 nM. (2) The induction of the activating group, such as the 4-methyl group on the phenyl ring of compound **2** (K_I_ value = 40.7 nM) led to compound **6**, with an increased hCA IX inhibitory activity (K_I_ value = 25.1 nM). (3) The introduction of the deactivating group on phenyl ring of compound **2**, such as the 4-bromo group, resulted in compound **3** with diminished hCA IX inhibition activity (K_I_ value of 75.2 nM); in contrast, the introduction of 4-fluoro/4-chloro groups produced compounds **4**–**5** with boosted the inhibitory potency of the hCA IX (K_I_ values of 8.0–16.5 nM). (4) *N*-propanamide **20**, with a K_I_ value of 18.6 nM, was powerful than the corresponding *N*-acetamide **11** with a K_I_ value of 66.8 nM. (5) The introduction of activating/deactivating groups on the phenyl ring of compound **2** (K_I_ value = 27.3 nM) yielded compounds **10**–**17** with reduced inhibitory activity (K_I_ values = 35.9–100.4 nM), except for compounds **12** and **16**, which had improved hCA IX inhibitory potency (K_I_ values = 12.7–24.1 nM). (6) Substitution of the phenyl group of compound **12** (K_I_ value = 12.7 nM) with a benzyl moiety resulted in compound **18,** which had significantly reduced hCA IX inhibitory activity (K_I_ value = 256.4 nM).

#### 3.3.4. SAR analysis for hCA XII inhibition

SAR analysis for hCA XII inhibition revealed several key factors. (1) 2-((2-Oxo-2-phenylethyl)thio)quinazolinone **2**, with a K_I_ value of 13.0 nM, was more potent than 2-((1-oxo-1-phenylpropan-2-yl)thio)quinazolinone **7** with a K_I_ value of 76.8 nM. (2) The introduction of a chloro/fluoro group at the phenyl ring, such as in compounds **4** and **5** (K_I_ values = 9.1–10.8 nM), improved the hCA XII inhibition activity and was similar to that of compound **2** (K_I_ value = 13.0 nM). (3) The unsubstituted *N*-acetamide, compound **8**, (K_I_ value = 2.4 nM) resulted in more powerful hCA XII inhibition than *N*-substituted amides, compounds **9**–**20**, (K_I_ values = 8.7–113.4 nM). (4) hCA XII inhibition of *N*-acetamide **11**, with a K_I_ value of 31.6 nM, was more powerful than that of the corresponding *N*-propanamide **20** with a K_I_ value of 66.7 nM. (5) The substitution of the phenyl group of *N*-(4-fluorophenyl)acetamide **12** (K_I_ value of 8.7 nM) with a benzyl moiety resulted in the *N*-(4-fluorobenzyl)acetamide **18**, with sharply reduced CA inhibitory activity (K_I_ value = 113.4 nM).

### 3.4. Molecular docking

#### 3.4.1. Molecular docking of compounds 17 and 20 with CA IX and CA XII isoenzymes

**Figure 2.**
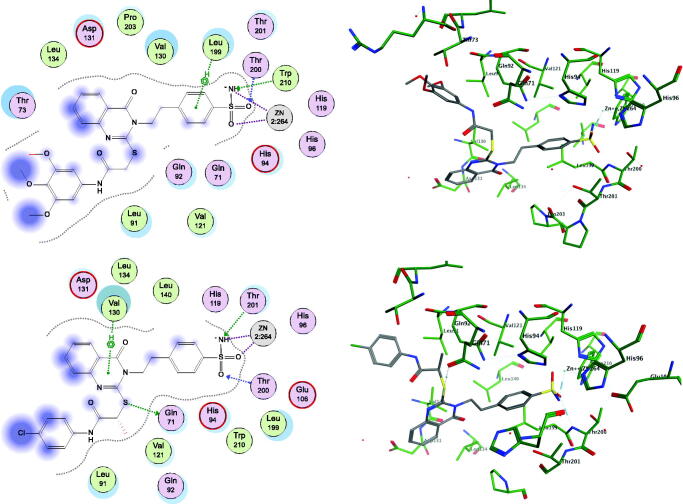
Docking modes of active compounds **17** and **20** in the binding pockets of CA isoenzyme IX (PDB 5FL4). Predicted binding mode of compounds **17** (2D and 3D in upper panel), and **20** (2D and 3D in lower panel) with the hCA-IX target.

**Figure 3.**
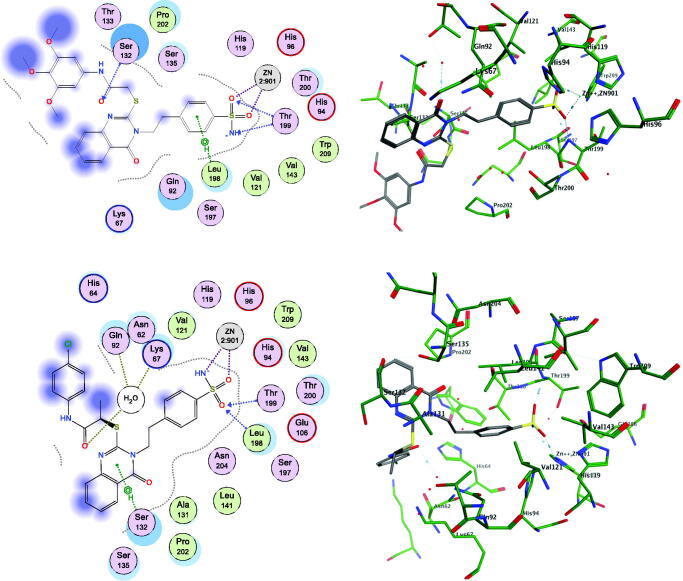
Docking modes of the active compounds **17** and **20** in the binding pockets of CA isoenzyme XII (PDB 1JCZ). Predicted binding mode of compounds **17** (2D and 3D in upper panel) and **20** (2D and 3D in lower panel) with hCA-XII target.

To further investigate the interactions between the selected active compounds **17** and **20** with the hCAs targets, we performed docking simulations into the binding pockets of the hCA isoforms, IX and XII, using the MOE Suite^65^ (data are summarised in Figures 2 and 3).

Both the compounds **17** and **20** were shown to directly interact with the zinc ion of CA IX and CA XII isoenzymes, *via* the sulphonamide anion of the active sites of both enzymes. However, the contributions of the quinazoline scaffold and the terminal bulky thioether fragments interaction are different, based on the CA isoform. In CA IX, the quinazoline ring of compound **20** interacts with the Gln71 residue through a stable hydrogen bond, and gets accommodated in the hydrophobic pocket lined by the Val121, Val130, Leu134, and Leu91 residues, thereby stabilising the binding (Figure 2, lower panel). In addition, the terminal *p*-chlorobenzamide fragment formed a hydrophobic interaction with the Leu91 residue (Figure 2, lower panel). In contrast, compound **17** was shown to bind similarly to the pocket of CA IX, except the unfavourable orientation of the quinazoline carbonyl moiety of compound **17** towards the hydrophobic pocket formed by Leu91 residue in CA IX (Figure 2, upper panel). Also, the benzamide core showed a polar-nonpolar interaction with the Leu91 and Thr73 residues, as the bulky side chain causes steric hindrance, inducing conformational changes in the bulky thioether tail and the quinazoline groups (Figure 2, upper panel). These differences in the binding of compounds **17** and **20** could be responsible for the observed differences in the K_I_ values of the two compounds for CA IX.

Results also showed different interactions between CA XII and compounds **17** and **20** (Figure 3). The carbonyl group on the quinazoline ring in compound **17** was stabilised by direct hydrogen bonding with the target residue Ser132 of CA XII (Figure 3, upper panel). In addition, the Lys67 residue showed favourable hydrophobic binding to the quinazoline core of compound **17**. The trimethoxybenzamide group of compound **17** was accommodated in the polar pocket of CA XII that included Ser132 and Thr133 residues (Figure 3, upper panel). The placement of compound **20** within the CA XII pocket was not favoured, particularly because the quinazoline ring of compound **20** was trapped between the polar pocket of CA lined by the Ser135, Gln92, and Ser132 residues (Figure 3, lower panel). Therefore, this interaction causes an energetically unfavourable change in the terminal benzamide and quinazoline scaffold of compound **20**, which could be responsible for the decreased inhibitory activity of compound **20** (Figure 3, lower panel).

#### 3.4.2. Molecular orbital analyses

**Figure 4.**
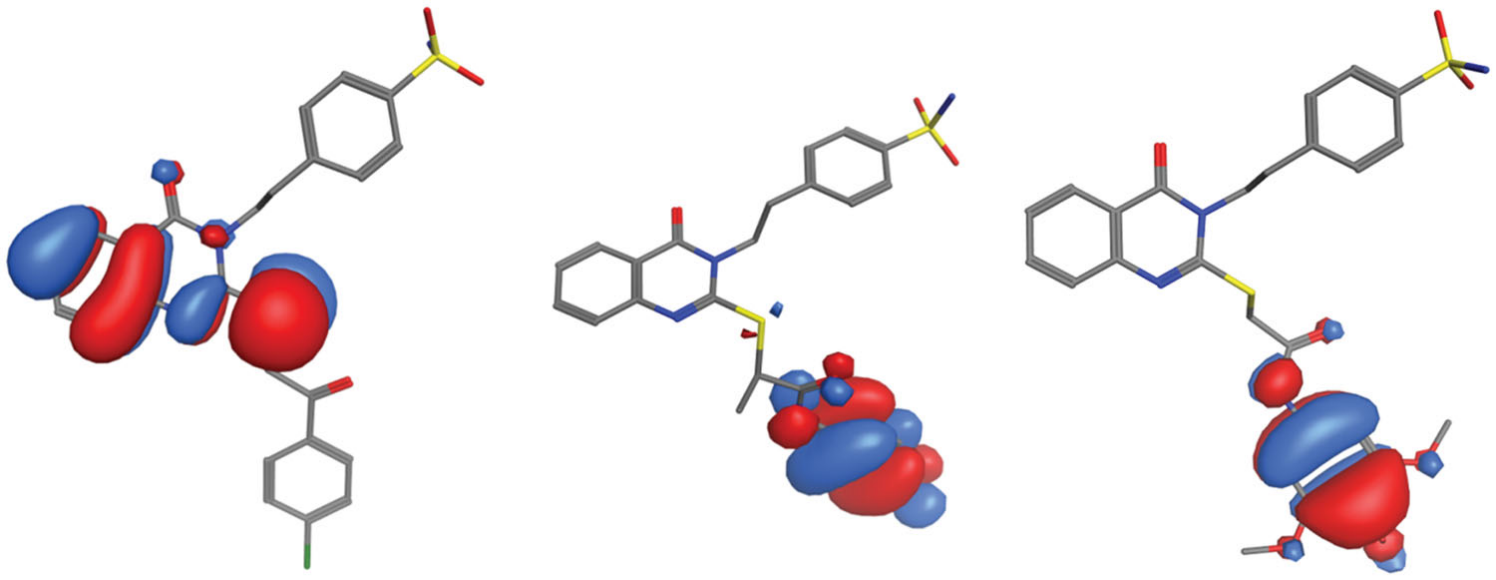
Molecular orbital spatial distribution and localisation for the HOMO and LUMO of three representative compounds, **4 (**left panel), **17 (**middle panel), and **20 (**right panel).

According to the frontier molecular orbital theory, HOMO and LUMO are the most important orbitals found in a molecule, as they can affect its biological activity, the molecular reactivity, the ionisation and the electron affinity^68–70^. The molecular orbital analysis of the representative compounds **4**, **17**, and **20** (Figure 4) as an active and selective derivatives was done by exploring their structure-selectivity relationship. The electron transition from HOMO to LUMO occurs freely when the energy gap is small. The HOMO-LUMO energy gap for the compounds **4**, **17**, and **20** was calculated to be −0.3125, −0.2834, and −0.28949 eV, respectively. The negative energy values are indicative of a stable structure and confirm the eventual charge transfer interactions. The distributions and energy levels of the HOMO-LUMO orbitals computed for the above-mentioned compounds are represented in Figure 4. HOMO and LUMO orbitals are mainly delocalised in the carbon and nitrogen of the quinazoline scaffolds and the sulphur ether atoms in the active compound **4**. While they are mainly delocalised in the S-linker of the benzamide moiety, ring substituents in the compounds **17** and **20** reverse their interactions with the enzyme isoforms. These results indicate that the affinity of the selective compounds for the CA IX and CA XII binding sites could be because of the involvement of the thioether moiety, and that the quinazoline moiety could mostly provide the structural basis and the lipophilic function, contributing strongly to their selectivity. In addition, the low HOMO-LOMO energy gap suggests that the molecules have high stability and are in their lowest energy conformation.

## 4. Conclusion

The CA inhibitory activity of 4-(2-(2-(substituted-thio)-4(3H)-quinazolinon-3-yl)ethyl)benzenesulfonamides (compounds **2**–**20**) towards the hCA I, II, IV, and IX isoforms was assessed and compared with acetazolamide (AAZ), a typical sulphonamide inhibitor. Of the different hCA isoforms, hCA I was effectively inhibited by the compounds **2** and **4–13**, with inhibition constant (K_I_) values in the range of 114.5–938.3 nM (AAZ: K_I_ value of 250.0 nM), while compounds **3** and **14–20** showed moderate to weak CA inhibitory activity with K_I_ values of 1447.0–5467 nM. Compounds **5**, **8**, **9**, **11**, **12**, and **20** were revealed to be effective hCA II inhibitors, with K_I_ values of 25.4–95.4 nM (AAZ: K_I_ value of 12.0 nM). Compounds **2**, **3**, **4**, **6**, **7**, **10**, **13**, **14**, **15**, and **16** showed modest to weak hCA II inhibitory activity with K_I_ values ranging between 116.2 and 1099.0 nM. Compounds **2–17** and **20** displayed potent hCA IX inhibitory activity with K_I_ values ranging from 8.0 to 100.4 nM compared to AAZ (K_I_ value of 25.0 nM), whereas compounds **18** and **19** showed modest hCA IX inhibitory activity with K_I_ values ranging between 256.4 and 145.1 nM, respectively. Ethylbenzenesulfonamide derivatives, **2**, **4**, **5**, **8**, **9**, **11**, **12**, **13**, **14**, **16**, and **17** showed potent hCA XII inhibitory activities with K_I_ values of 2.4–49.1 nM compared to AAZ (K_I_ value of 5.7 nM), whereas compounds **3**, **6**, **7**, **10**, **15**, **18**, **19**, and **20** showed moderate hCA XII inhibitory activities with K_I_ values of 59.7–113.4 nM. Compounds **2** and **4** showed characteristic effective and selective antitumor (hCA IX and hCA XII) carbonic anhydrase inhibitory activity with K_I_ values (compound **2**; 40.7 and 13.0 nM) and K_I_ values (compound **4**; 8.0, 10.8 nM). Compounds **2–7** showed high selectivity ratios for the inhibition of hCA IX over hCA I (15.0–95.0) and hCA IX over hCA II (2.3–23.0), while selectivity ratios of hCA XII over hCA I (5.5–70.0) and hCA XII over hCA II (1.4–17.0). Compounds **4** and **5** displayed selective inhibitory activity towards hCA IX over hCA I with selectivity ratios of 95.0 and 24.0 respectively, and hCA IX over hCA II with selectivity ratios of 23.0 and 5.8 respectively, as well as, selective inhibitory activity for hCA XII over hCA I and hCA XII over hCA II (selectivity ratios of 70.0, 44.0 and 17.0, 10.0, respectively). Compounds **12**–**17**, and **19**–**20** exhibited selective inhibitory activities towards hCA IX over hCA I and hCA IX over hCA II (selectivity ratios of 23.0–195.0 and 3.2–19.0, respectively). In addition, compounds **8**, **12**, **14**–**17**, and **19** showed selective inhibitory activity towards hCA XII over hCA I and hCA XII over hCA II (selectivity ratios of 48.0–158.0 and 5.4–31.0, respectively). Docking study of the selective derivatives, compounds **17** and **20**, with the hCAs revealed consistent interactions, particularly selectivity-oriented hydrophobic and aromatic interactions through the S-alkyl substituent.

## Supplementary Material

Supplemental Material
