# Supplementary figures and images for: S-substituted 2-mercaptoquinazolin-4(3H)-one and 4-ethylbenzensulfonamides act as potent and selective human carbonic anhydrase IX and XII inhibitors
Source: J Enzyme Inhib Med Chem. 2020 Mar 19;35(1):733–43. doi: 10.1080/14756366.2020.1742117 (PMC7144323; doi:10.1080/14756366.2020.1742117)

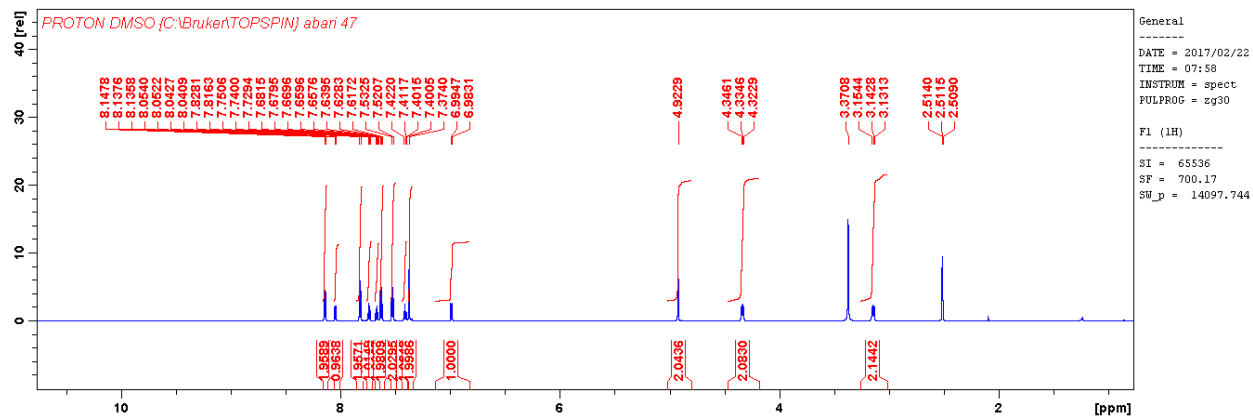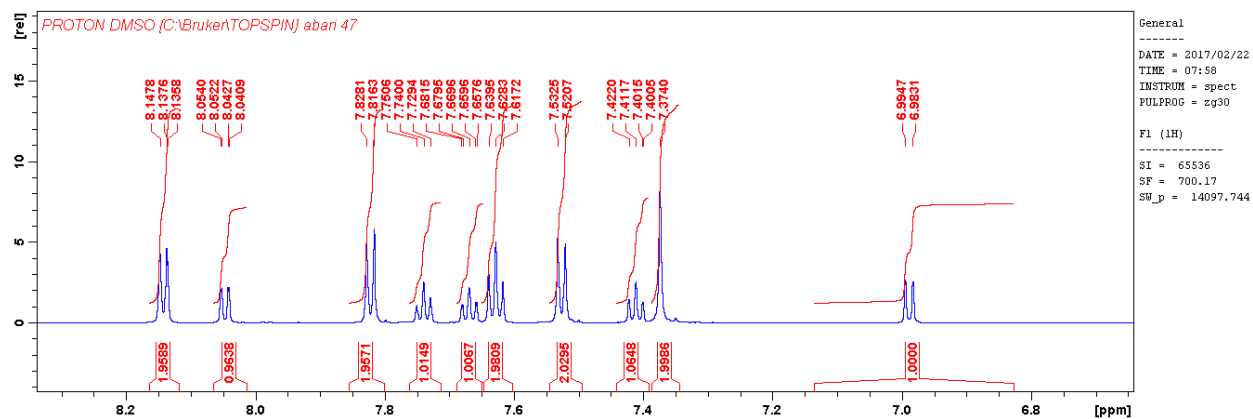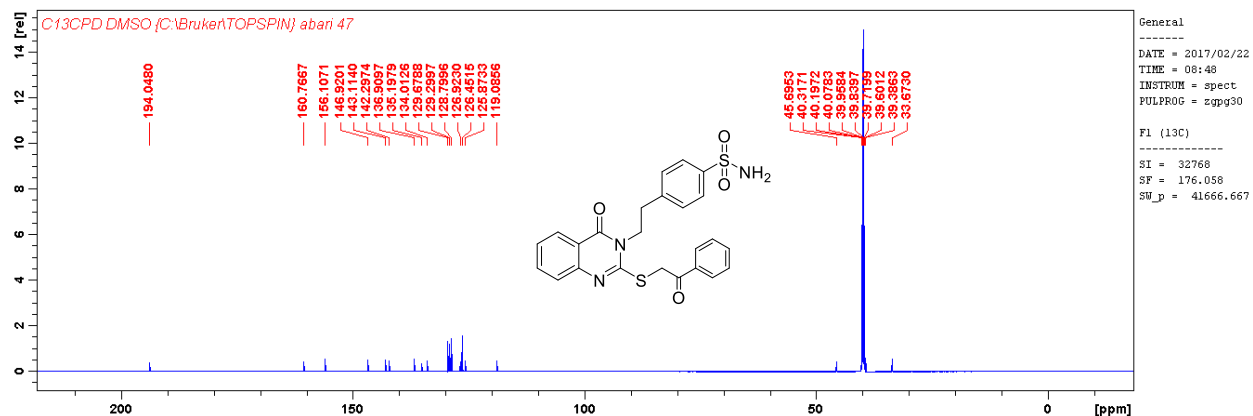

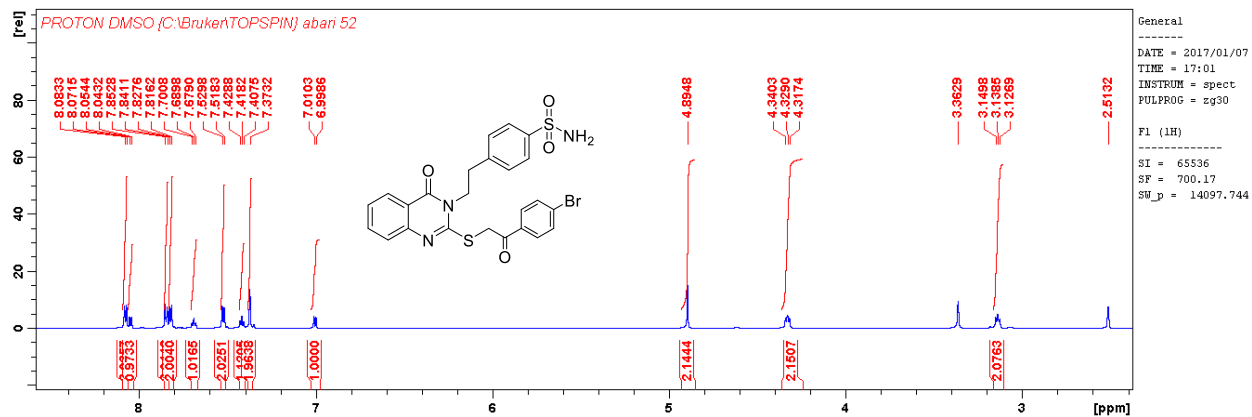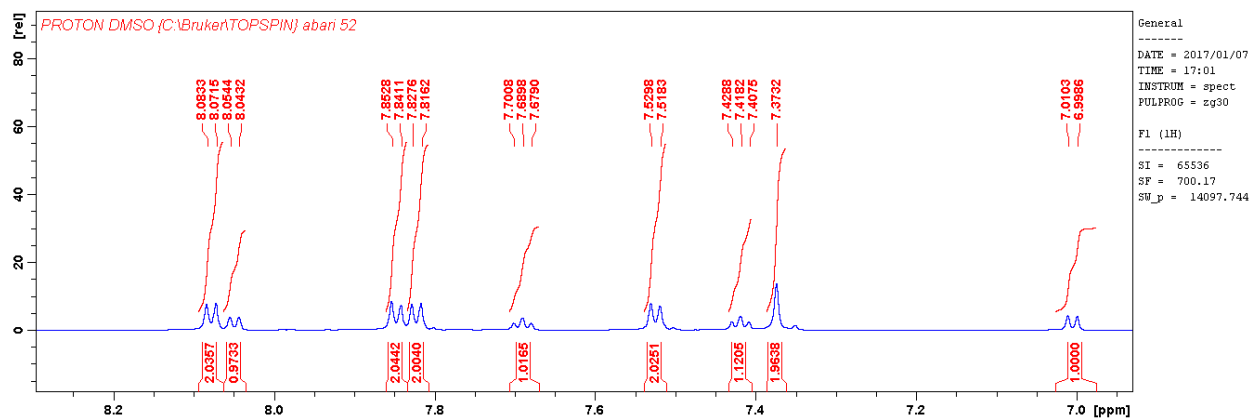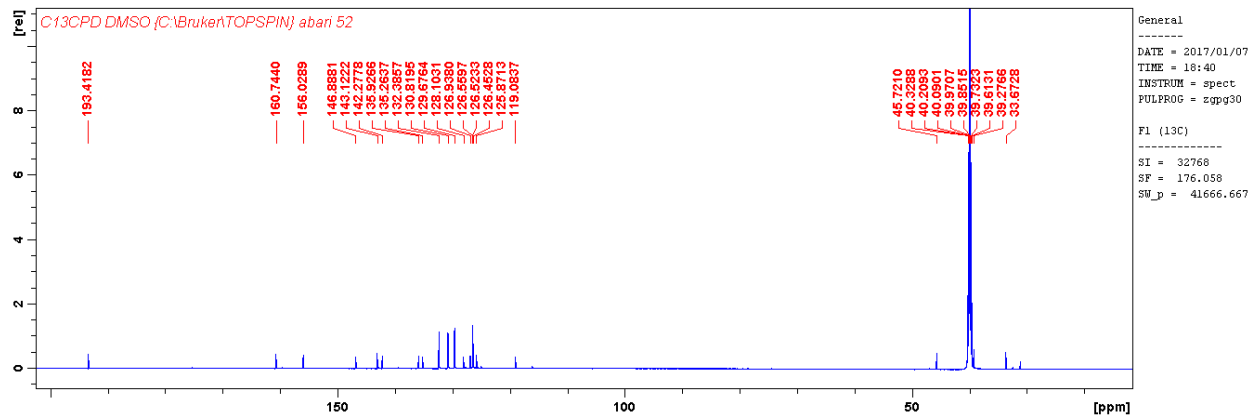

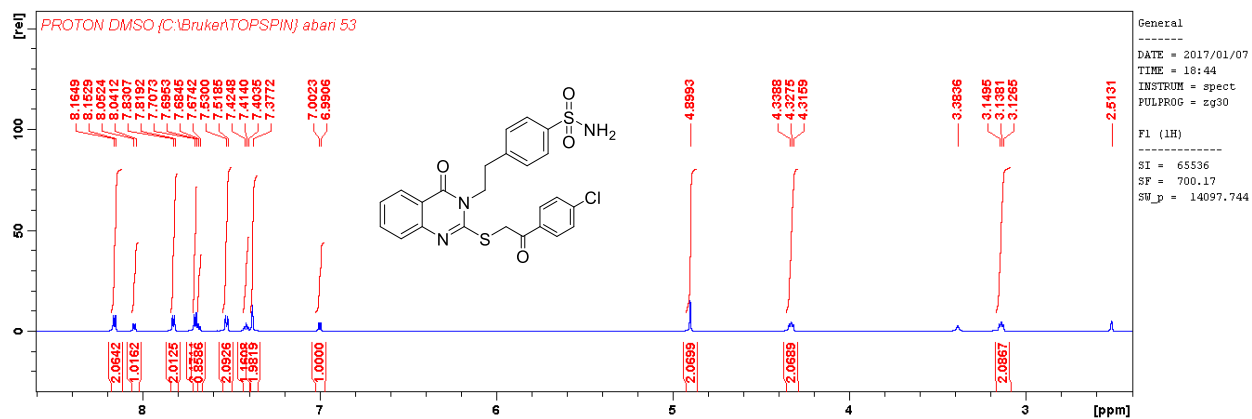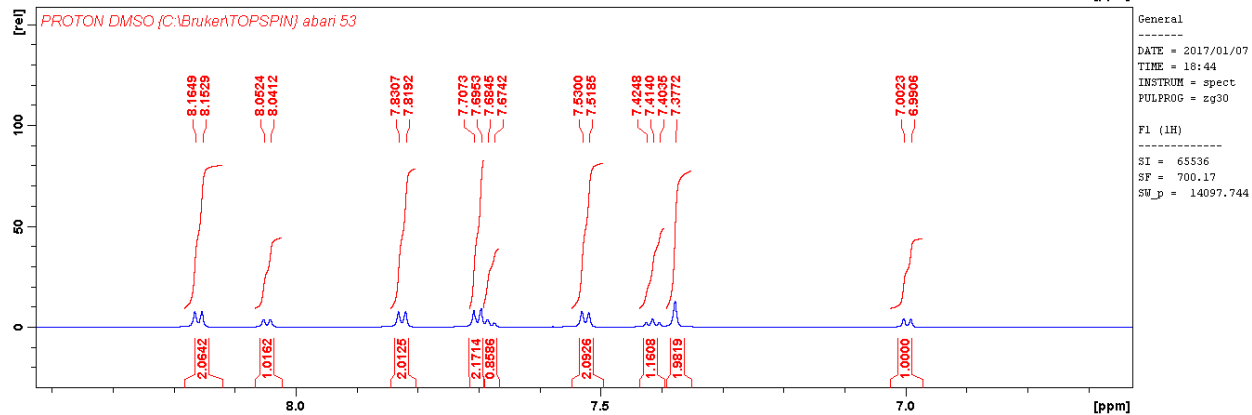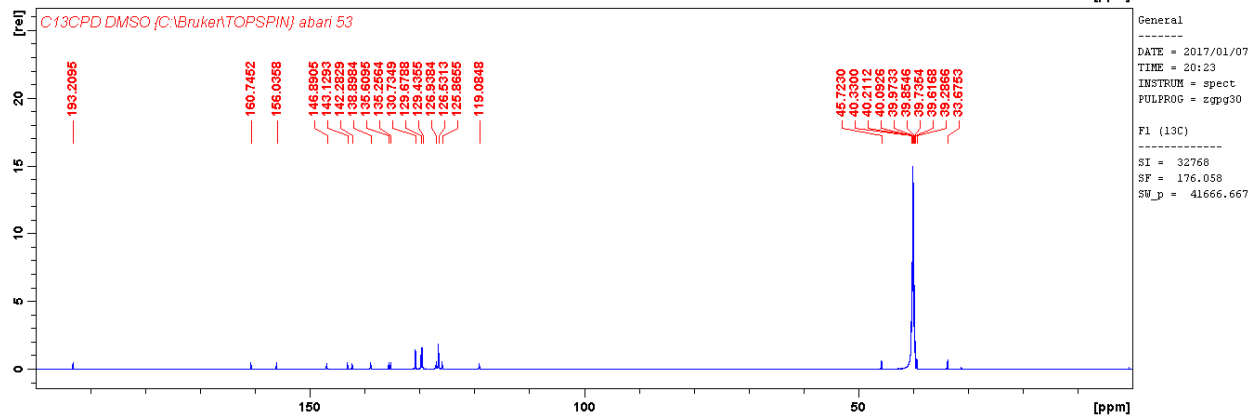

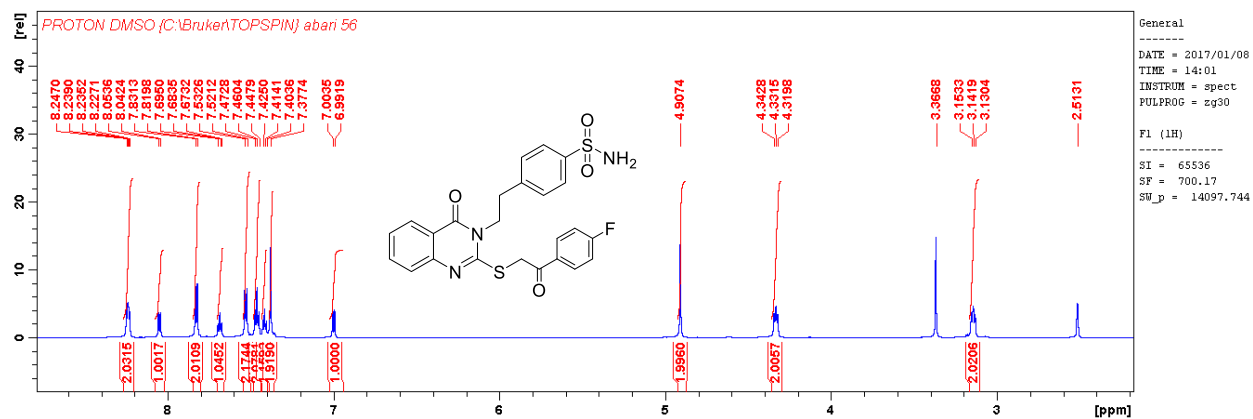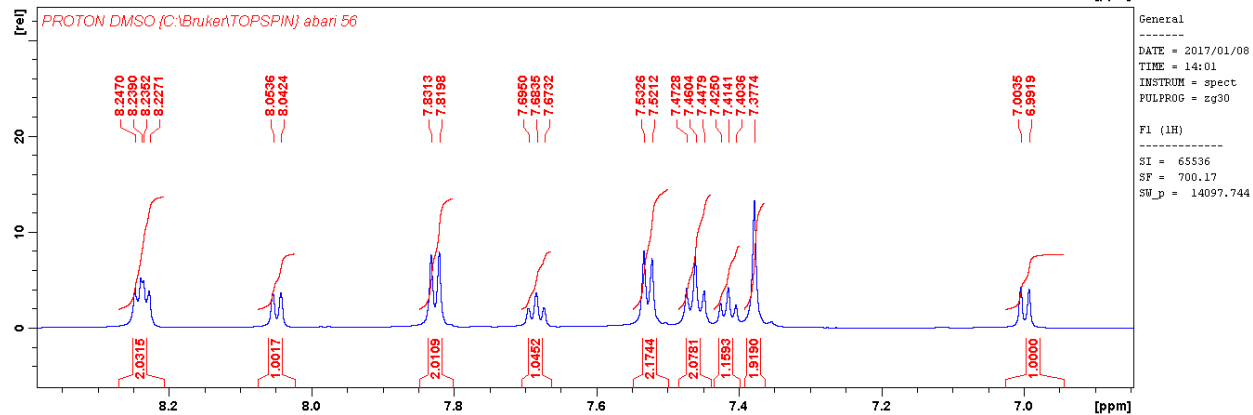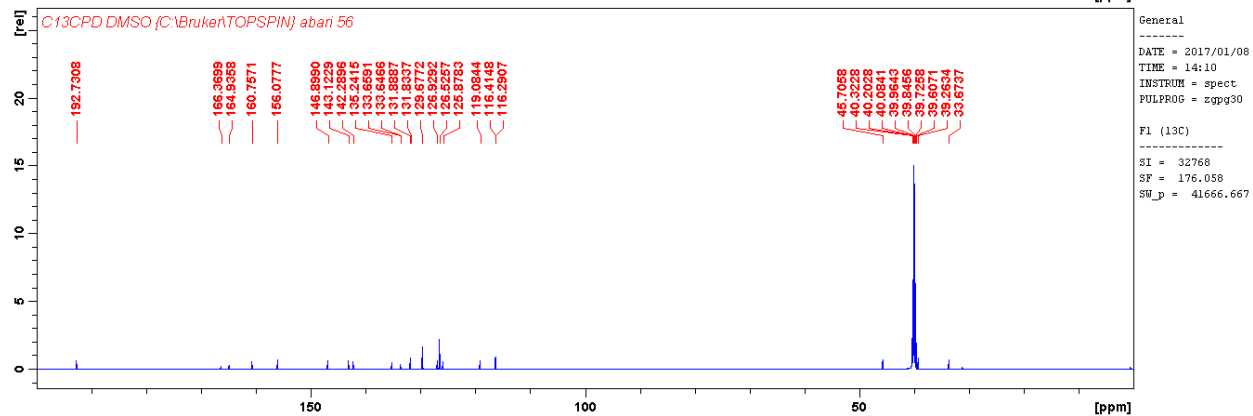

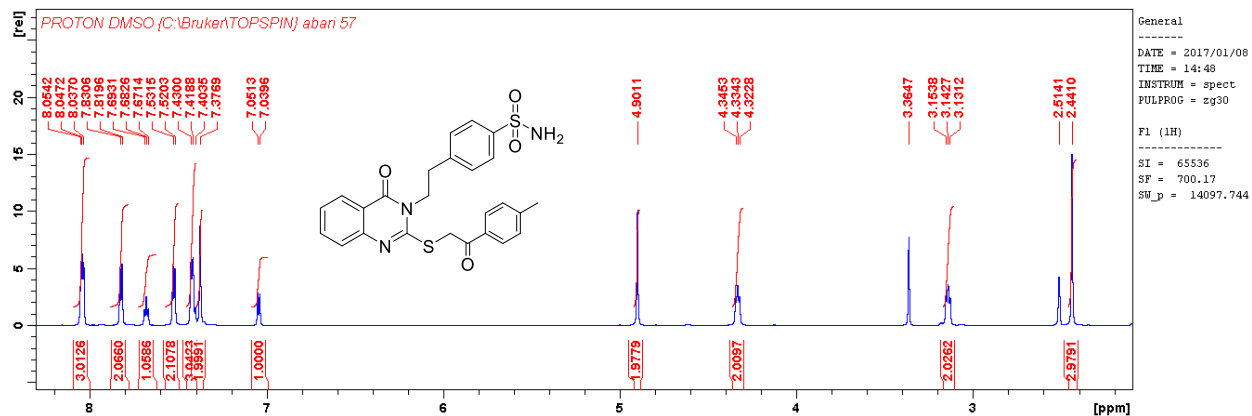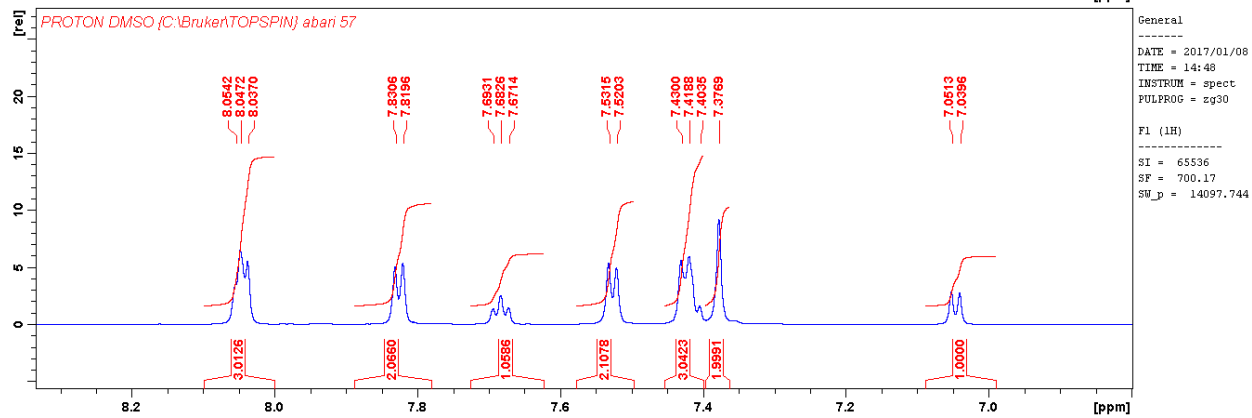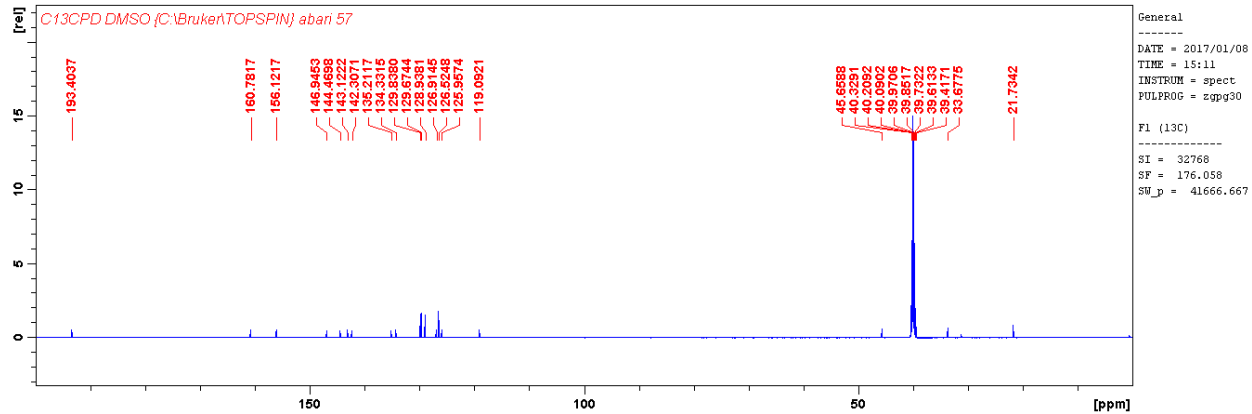

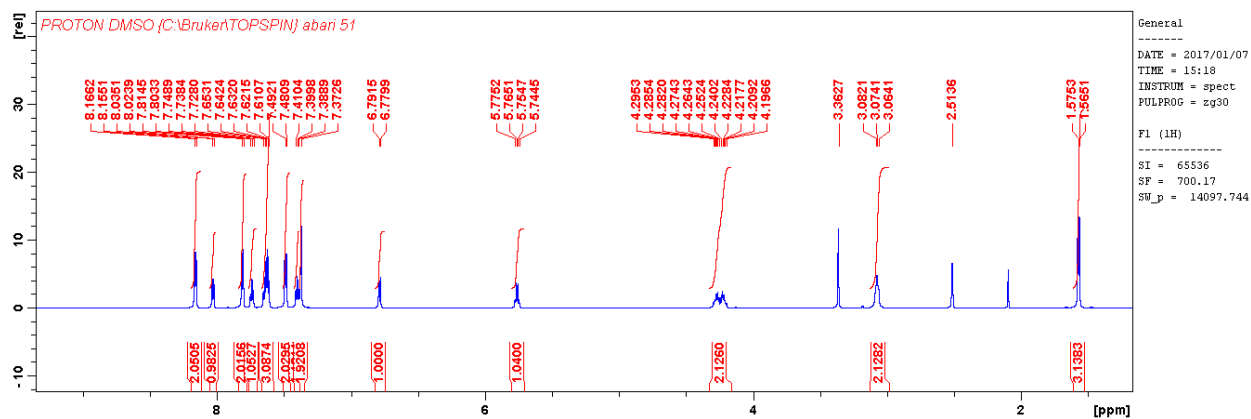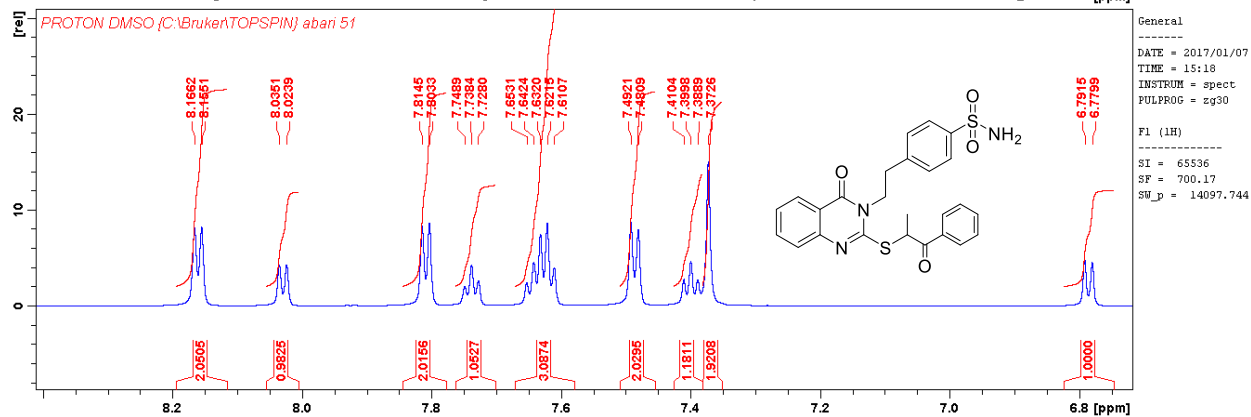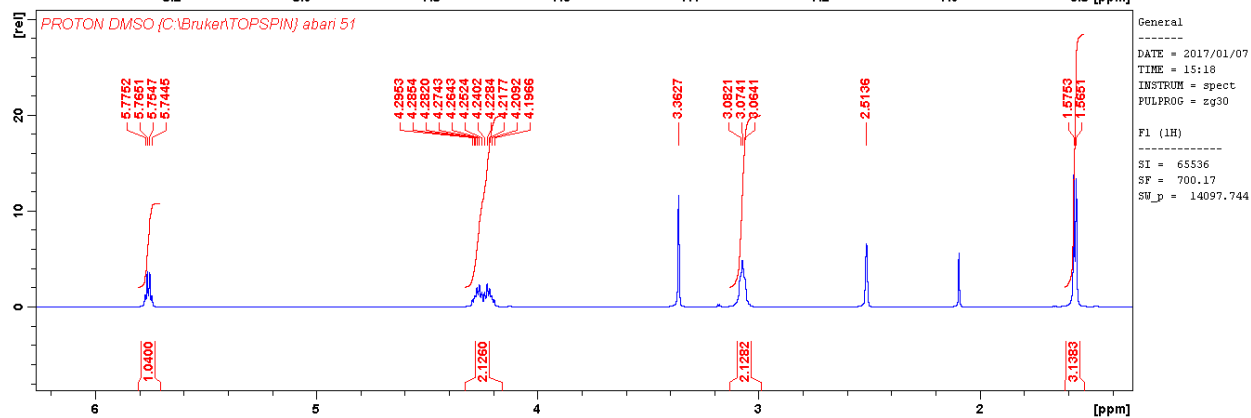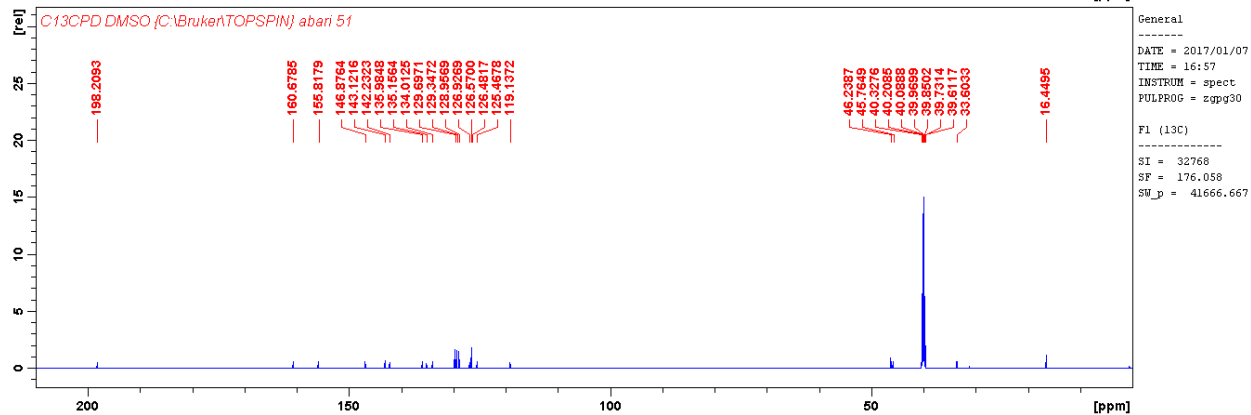

Supplement: Supplemental Material [file IENZ_A_1742117_SM5356.pdf]
